# Engagement, efficacy, and experiences of psychotherapy for perinatal populations with depression and anxiety during the COVID-19 pandemic

**DOI:** 10.3389/fpsyt.2025.1686719

**Published:** 2026-01-22

**Authors:** Holly A. Krohn, Sandeep Shelly, Jamie E. Gibori, Nicole Andrejek, Laura La Porte, Andrea S. Lawson, Aditya Anand, Tara S. Berenbaum, Cindy-Lee Dennis, Adrienne Griffen, Zoe Lea, Robert G. Maunder, Samantha Meltzer-Brody, Simone N. Vigod, Richard K. Silver, Daisy R. Singla

**Affiliations:** 1 Department of Psychiatry, School of Medicine, University of North Carolina, Chapel Hill, NC, United States; 2 Campbell Family Mental Health Research Institute, Centre for Addiction and Mental Health, Toronto, ON, Canada; 3 Department of Obstetrics and Gynecology, Endeavor Health, Evanston, IL, United States; 4 Lunenfeld-Tanenbaum Research Institute, Sinai Health, Toronto, ON, Canada; 5 Department of Psychiatry, Temerty Faculty of Medicine, University of Toronto, Toronto, ON, Canada; 6 Lawrence S. Bloomberg Faculty of Nursing, University of Toronto, Toronto, ON, Canada; 7 Maternal Mental Health Leadership Alliance, Arlington, VA, United States; 8 Department of Sociology, Faculty of Social Sciences, McMaster University, Hamilton, ON, Canada; 9 Department of Psychiatry, Women’s College Hospital, Toronto, ON, Canada; 10 Department of Obstetrics & Gynecology, Pritzker School of Medicine, University of Chicago, Chicago, IL, United States

**Keywords:** COVID-19, perinatal depression, perinatal anxiety, telemedicine, public health

## Abstract

**Background:**

Engagement in psychotherapy is essential for achieving effective mental health outcomes, yet maintaining participation can be challenging—especially during significant disruptions such as the COVID-19 pandemic. The SUMMIT Trial evaluated a brief behavioral activation treatment for perinatal depression and anxiety, comparing telemedicine with in-person psychotherapy. Due to pandemic-related disruptions, in-person randomization was suspended twice, dividing participants into peak and non-peak COVID-19 timeframes.

**Methods:**

This secondary mixed-methods analysis examined enrollment, retention, satisfaction, and depressive and anxiety symptoms between peak (March 2020-July 2021 & Jan-April 2022) and non-peak COVID-19 periods (Jan-March 2020, Jul 2021-Jan 2022, April 2022-Sep 2023). During peak-COVID-19, participants received telemedicine-only; otherwise, they were randomized to telemedicine or in-person treatment. T-tests compared symptom scores and enrollment rates; chi-square and logistic regression analyzed retention and satisfaction. Qualitative data underwent thematic analysis.

**Results:**

Of 1230 participants, 597 (48.5%) enrolled during peak-COVID and were randomized to telemedicine, while 693 (56.3%) were randomized to telemedicine or in-person treatment. Enrollment (25.4 vs. 21.8 participants/month, *p* = 0.250), retention (98.12% vs. 98.42%, *p* = 0.689) and satisfaction (CSQ-8: 3.45 vs. 3.39, *p* = 0.174) did not differ significantly. No differences were observed in depressive or anxiety symptoms at baseline (EPDS: 15.85 vs. 15.72, *p* = 0.546; GAD-7: 11.70 vs. 11.96, *p* = 0.3670) or at 3 months (EPDS: 9.09 vs. 9.09, *p* = 0.980; GAD-7: 6.42 vs. 6.38, *p* = 0.907).

**Conclusions:**

Engagement, efficacy, and experience were comparable across pandemic phases, highlighting the feasibility of telemedicine-based adaptations in the midst of public health crises.

## Introduction

The COVID-19 pandemic placed an unprecedented strain on healthcare systems, significantly disrupting access to medical care, including mental health services (1). In the perinatal population, the inability to see patients in person further constrained treatment availability (2) and mental health treatments were deprioritized in deference to lifesaving care (3). These challenges—exacerbated by the pandemic—underscore the urgent need to create patient-centered models of care during public health crises and to assess any therapeutic limitations associated with modifying usual care standards.

The Scaling Up Maternal Mental healthcare by Increasing access to Treatment (SUMMIT) trial aimed to aimed to compare telemedicine vs. in-person delivered psychotherapy from specialist (e.g., licensed clinicians) and non-specialist (e.g., nurses, midwives) providers (4, 5). The trial was conducted throughout the COVID-19 pandemic between January 2020 and October 2023 and, required significant modification of its original study design due to pandemic-related factors. The operational changes included strengthening clinical referral pathways, transitioning research staff to remote work with secure server access, and implementing secure telemedicine platforms for treatment delivery and storage of encrypted session recordings (4). The in-person study arms were suspended twice during peak infection prevalence corresponding to the onset of the pandemic through the highly infectious Delta variant, and then again during the height of the Omicron variant when case prevalence increased dramatically. The primary analysis at 3-month post-randomization demonstrated that task-sharing and telemedicine were non-inferior to specialist-delivered, in-person treatment for perinatal depression and anxiety outcome (5). While there had been no planning to accommodate the pandemic, a modified analytical plan was put into place along with in-person restrictions to accommodate the COVID-19 disruptions (ClinicalTrials.gov: NCT04153864).

Similar to all psychotherapy trials, the SUMMIT Trial relied on participant engagement for both scientific rigor and clinical application (6–8). Key variables related to participant engagement included rates of participant enrollment, retention, and satisfaction. *Enrollment rates* reflected an intervention’s reach, while *retention rates* were a measure of intervention feasibility and engagement. Finally, *participant satisfaction ratings* provided insight into the actual experience of treatment and its perceived effectiveness. These factors may be particularly relevant in task-sharing models, where non-specialist providers (individuals with no formal training or mental healthcare background) deliver care, as well as in telemedicine which requires sustained remote engagement to achieve effectiveness (9). The concurrence of the pandemic may impact all of these factors as well as treatment effectiveness.

Thus, the current study aimed to assess the impact of COVID-19-related adaptations within the trial on participant engagement, treatment efficacy and patient experience during peak and non-peak COVID-19 periods. In this study, peak-COVID periods are defined as a phase during which randomization and treatment delivery were restricted to telemedicine as a result of increased COVID cases in the general population often leading to lockdowns (March 2020-July 2021; January 2022-April 2022). The remainder of the trial period was classified as non-peak.

This exploratory study to the SUMMIT Trial had three primary aims:

1. To evaluate patient engagement (rates of recruitment, retention and satisfaction scores) in perinatal depression treatment during the COVID-19 pandemic, by comparing enrollment and retention rates across peak and non-peak pandemic periods.
2. To assess treatment efficacy of brief behavioral activation (BA) delivered via telemedicine during peak versus non-peak COVID-19 periods, as measured by changes in patient symptoms of depression and anxiety from enrollment to 3-month measurement.
3. To explore patient experience during these periods using both quantitative satisfaction data and qualitative analysis of reported barriers and facilitators to care.

## Methods

### Design and setting

This mixed methods study is a secondary analysis of the Scaling Up Maternal Mental Healthcare by Increasing Access to Treatment (SUMMIT) trial, which was designed to evaluate the non-inferiority of Behavioral Activation (BA) delivered by specialist vs. non-specialist providers and via telemedicine vs. in-person modalities for perinatal depression and anxiety treatment. The design of the SUMMIT trial (ClinicalTrials.gov: NCT04153864) has been described in detail elsewhere (4, 5). The study recruited participants from university-affiliated healthcare settings, clinical sites, and obstetric and family medicine clinics across Chapel Hill, North Carolina, USA; Evanston/Chicago, Illinois, USA; and Toronto, Canada.

### Participants

Recruitment began in early 2020, just prior to the COVID-19 pandemic, and continued through late 2023. A total of 1230 pregnant and postpartum women were enrolled across sites. Participants were introduced to the study by clinical providers or trained research assistants. Inclusion and exclusion criteria for the larger trial are described elsewhere (4). All participants provided written or electronic informed consent via REDcap™ before enrollment. Ethical approval was obtained from the following institutional review boards (IRBs): UNC Biomedical IRB (19-1786); Endeavor Health IRB (EH18-129); and Clinical Trials Ontario (1895).

**Figure 1:**
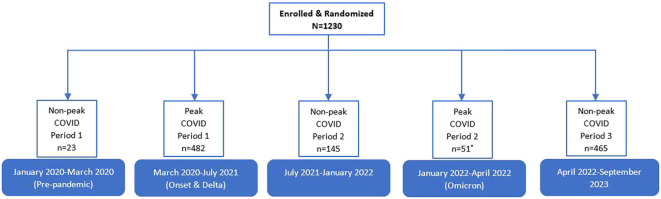
Distribution on Peak and Non-peak COVID-19 cases during the SUMMIT trial (January 2020-September 2023). Due to site-specific differences in response to the Omicron variant, 64 additional participants were categorized as “Non-Peak COVID” from January 2022-April 2022.

Between March 2020–July 2021 and January 2022–April 2022, a COVID-restricted randomization strategy was employed, where all participants received telemedicine-only treatment but were still able to be randomized to either specialist or non-specialist care. These periods are referred to as “peak-COVID-19.” In addition, any patients who were randomized 1:1 to the two telemedicine arms were grouped into the peak-COVID category (**Figure 1**). During non-peak COVID-19, participants were randomized to either in-person or telemedicine treatment and as the pandemic resolved, a weighted randomization strategy favoring in-person care was employed to partially restore the balanced allotment that was originally planned (5). Qualitative interview participants during peak COVID-19 completed all BA sessions by telemedicine, while participants interviewed in the non-peak period completed BA either in-person or by telemedicine.

### Intervention

Participants received up to 8 sessions of a brief, manualized BA intervention delivered by a specialist provider (SP) (e.g., psychologists, psychiatrists, social workers) or a trained non-specialist provider (NSP) (e.g., nurses, midwives, doulas). Treatment was delivered either in-person or by video via a HIPAA/PHIPA-compliant telemedicine video platform, such as Zoom™ or WebEx™, in a setting of the participant’s choice. During peak-COVID periods, all BA sessions were conducted via telemedicine, while during non-peak periods, participants were randomly assigned to receive in-person or telemedicine-based treatment.

BA is a transdiagnostic treatment that is effective for depressive and anxiety symptoms (10–13), including for ethnically-diverse perinatal populations (14, 15). The intervention focuses on increasing engagement in enjoyable and meaningful activities that align with one’s values, aiming to reduce mental health symptoms (16, 17). Further details on the intervention are provided elsewhere (4, 18–20). The BA manual used in the SUMMIT Trial is now freely available (www.thesummittrial.com).

### Demographic and clinical characteristics

Demographic and clinical data were collected at baseline and 3 months post-randomization using REDCap™. Demographic variables included participant age, perinatal period (pregnancy or postpartum), race/ethnicity, gender identity, sexual orientation, marital status, employment status, education level, and treatment preferences. Clinical data included psychotropic medication use and pregnancy-related conditions. Participants were categorized into white and racial/ethnic minority groups based on self-report. The number of completed BA sessions was recorded at 3 months post-randomization.

### Measures

#### Enrollment and retention

Enrollment rate was defined as the number of participants randomized per month; retention rate was defined as the number of individuals who (a) remained in the trial before starting BA and (b) remained during the treatment phase. A robust retention strategy was implemented throughout the study to maximize participant retention and patient-centeredness (21). These efforts included pre-notification emails, follow-up messages via email, phone, and text, and routine updates to participant contact information and training all research personnel in common therapeutic skills such as demonstrating empathy and a non-judgmental stance (5). Participant engagement was further supported through quarterly newsletter reminders, in addition to the information about the study progression. As a result, the study achieved a low dropout rate.

#### Client Satisfaction Questionnaire-8

The Client Satisfaction Questionnaire-8 (CSQ-8) is an 8-item Likert scale designed to gauge satisfaction with psychotherapy services and was completed by the participants at 3 months post-randomization. It assesses the quality of treatment, the likelihood of recommending the service to others, and the probability of seeking future support or returning for more treatment. Responses are averaged on a scale from 1 to 4, with higher scores reflecting greater satisfaction. The CSQ-8 is well-regarded for its reliability and has been linked to symptom improvement (22). It has been validated in studies involving perinatal populations (23) as well as diverse ethnic groups (24).

#### Symptom scores

Symptom scores were collected at baseline and 3 months post-randomization. Depressive and anxiety scores were measured by the 10-item Edinburgh Postnatal Depression Scale (EPDS) and the 7-item Generalized Anxiety Disorder-7 (GAD-7), respectively. The EPDS is a 10-item, Likert scale (range 0-30) that is highly sensitive to change and is the most common measure of perinatal depressive symptoms in clinical and research settings (25–27). The GAD-7 is a 7-item scale (range 0–21) with good internal consistency, reliability, validity and is sensitive to change (28, 29).

#### Qualitative interviews

Qualitative data were collected over two time periods: 1) during the peak COVID-19 period of September to December 2020 and 2) during the non-peak period of August to October 2022. Qualitative researchers conducted semi-structured, individual interviews with participants across all three SUMMIT study sites. Qualitative interviews were extended to a subset of consenting participants to further explore perinatal perspectives. Participants who completed a minimum of 5 treatment sessions and consented to a qualitative interview were randomly selected and the sample size was determined through maximum saturation methods. Interviews were conducted using a HIPAA/PHIPA-compliant version of Zoom™. Each interview lasted 40–60 minutes. In the interviews, all participants were asked open-ended questions about their perspectives of facilitators and barriers to positive care experiences, in which themes related to facilitators and barriers were endorsed spontaneously by participants (see **Appendix 1** in **Supplementary Materials** for interview questions).

#### Statistical methods

All baseline variables of interest were summarized using means and two-sided 95% Confidence Intervals (CIs) for continuous measures, and percentages for categorical measures. Differences in baseline characteristics and symptom scores between peak and non-peak COVID-19 time periods were evaluated using a two-sample t-test for continuous variables and a chi-square test for categorical variables.

To compare participant enrollment rates during peak and non-peak periods, the monthly enrollment rate was analyzed using a two-sample t-test. Retention rates before and during treatment across peak and non-peak COVID-19 periods were assessed with a chi-square test of independence. Logistic regression was employed to examine the relationship between retention rates and COVID-19 periods, while controlling for treatment modality and race/ethnicity.

Client satisfaction during peak and non-peak COVID-19 periods was compared using a two-sample t-test. Imputation methods were applied to incorporate the missing data for individuals who had completed at least one treatment session but did not respond to the CSQ-8 measure. Multiple linear regression was utilized to examine the relationship between CSQ-8 and COVID-19 periods, controlling for patient’s household income, perinatal status (pregnant or postpartum), treatment modality and race/ethnicity.

A subgroup analysis was conducted by excluding in-person participants from the non-peak period to compare the telemedicine-only treatment delivery between peak- and non-peak periods. In this analysis, linear and logistic regression models included only race/ethnicity as a covariate. The selection of covariates was based on descriptive statistics indicating significant differences between groups, and variables with high inter-correlation were excluded from the analysis. For retention rates, a subgroup analysis was not performed as during the non-peak period, both in-person and telemedicine enrollment occurred. A subgroup analysis excluding in-person individuals in the enrollment numbers introduces selection bias and does not account for the context of the entire enrollment process.

All statistical analyses were conducted using SAS 9.4 software.

#### Qualitative analysis

Using established methodologies, qualitative data were coded using NVivo™ and were analyzed using thematic analysis (21, 30). The interview guide was used to develop an initial coding index. Coding was done through a 3-step process: a line-by-line coding stage, a follow-up coding stage in which new themes emerged and were synthesized, and a final coding stage in which all codes were organized thematically. Inter-rater reliability, assessed using Cohen’s Kappa (κ), demonstrated excellent agreement between coders (κ=0.95). Descriptive statistics (means, ranges, and frequencies) were calculated to summarize qualitative findings.

## Results

### Baseline characteristics

Among 1230 participants, 597 were peak participants (i.e., randomized to telemedicine arms only) and 633 were non-peak participants (i.e., randomized to telemedicine and in-person arms). The distribution between pregnant and postpartum participants was nearly balanced in both groups (**Table 1**). Peak participants were significantly younger (32.79 vs. 33.64 years, *p* = 0.003) and more likely to identify as white (55.77% vs. 45.77, *p =* 0.0009) than non-peak participants. Peak period participants completed significantly more sessions (7.04 (SD = 1.83) vs. 6.64 (SD = 2.22), *p =* 0.001) than non-peak participants, likely owing to exclusive telemedicine delivery. Participants’ pre-randomization preference for telemedicine was also higher during the peak period (65.50% vs. 59.57%, *p =* 0.009). Two individuals endorsed COVID-19 exposure at baseline during the peak time frame, compared to 20 during the non-peak time frame (Peak: n = 2, 0.51% vs. Non-peak: n = 20, 5.03%, *p* = 0.002), perhaps related to less vigilance among the public when infection prevalence was lower. We also examined baseline characteristics among participants retained at 3 months post-randomization (n = 1098), which demonstrated similar distributions and between-group differences as the full (n = 1230) cohort (**Supplementary Table 1**). In addition, these characteristics did not differ among participants who dropped out of the study (n = 132; **Supplementary Table 2**).

**Table 1:** Baseline participant characteristics categorized by peak and non-peak COVID-19 timeframes.

Variable [total respondents]	Overall (N = 1230)	Peak (n=533)	Non-peak (n=697)	*t-value/χ^2^*
Age (years) [1226], mean (95% CI)^a^	33.27 (33.00-33.55)	32.79 (32.37-33.21)	33.64 (33.28-34.01)	3.01^**^
Perinatal period [1230]				
Pregnant	618 (50.24)	277 (51.97)	341 (48.92)	1.12
Postpartum	612 (49.76)	256 (48.03)	356 (51.08)	
Race (White vs. BIPOC^^^) [1226] ^a^				
White	614 (50.08)	295 (55.77)	319 (45.77)	14.06^**^
Racial and ethnic minorities	578 (47.15)	217 (41.02)	361 (51.79)	
Prefer not to answer	34 (2.77)	17 (3.21)	17 (2.44)	
Born in country of your current residence? [1226]^a^	860 (70.15)	380 (71.83)	480 (68.87)	5.38
Health Benefits Access^Δ^ [948]^b^	654 (68.99)	198 (72.26)	456 (67.66)	2.64
Marital status [1226]^a^				
Married or Stable relationship	1052 (85.81)	460 (86.96)	592 (84.94)	1.32
Single	94 (7.67)	36 (6.81)	58 (8.32)	
Dating or Uncommitted relationship	41 (3.34)	17 (3.21)	24 (3.44)	
Other	21 (1.71)	8 (1.51)	13 (1.87)	
Prefer not to answer	18 (1.47)	8 (1.51)	10 (1.43)	
Gender identity [1173]^b^				
Female	1168 (99.57)	476 (99.58)	692 (99.57)	0.83
Sexual orientation [1173]^b^				
Straight/Heterosexual	1073 (91.47)	438 (91.63)	635 (91.37)	2.46
Bisexual	71 (6.05)	25 (5.23)	46 (6.62)	
Other	7 (0.60)	4 (0.84)	3 (0.43)	
Prefer not to answer	22 (1.88)	11 (2.30)	11 (1.58)	
Highest level of education [1226]^a^				
University degree	859 (70.07)	373 (70.51)	486 (69.73)	3.04
College/Trade School	216 (17.62)	89 (16.82)	127 (18.22)	
High School or less	141 (11.50)	65 (12.29)	76 (10.90)	
Prefer not to answer	10 (0.82)	2 (0.38)	8 (1.15)	
Employment [1226]^a^				
Full-time	484 (39.48)	204 (38.56)	280 (40.17)	6.02
Maternity leave	353 (28.79)	142 (26.84)	211 (30.27)	
Part-time	111 (9.05)	47 (8.88)	64 (9.18)	
Not employed	260 (21.21)	129 (24.39)	131 (18.79)	
Other	18 (1.47)	7 (1.32)	11 (1.58)	
Nulliparous[1226]^a^	668 (54.49)	293 (55.39)	375 (53.80)	3.24
COVID Exposure [1064]^b^	31 (2.91)	2 (0.51)	29 (4.31)	155.15^***^
Self-Reported History of Depression/Anxiety [1226]^a^	1051 (85.73)	460 (86.96)	591 (84.79)	1.88
Related to the current Baby	321 (30.54)	139 (30.22)	182 (30.80)	1.15
Dosage [1119]	6.82 (6.70-6.94)	7.04 (6.88-7.20)	6.64 (6.47-6.82)	-3.22^**^
Psychotropic medication Use at Baseline [288]^c^	288 (100)	133 (100)	155 (100)	–
Pregnancy conditions during current pregnancy [1075] ^b^				
Preeclampsia	52 (4.84)	18 (4.49)	34 (5.04)	2.31
High blood-pressure	127 (11.81)	46 (11.47)	81 (12.02)	0.31
Gestational diabetes	121 (11.26)	46 (11.47)	75 (11.13)	0.07
Preterm labor	69 (6.42)	25 (6.23)	44 (6.53)	0.56
Treatment preference expressed at baseline [1203]^b^				
Telemedicine	747 (62.09)	336 (65.50)	411 (59.57)	9.49^**^
In-Person	190 (15.79)	62 (12.09)	128 (18.55)	
No Preference	266 (22.11)	115 (22.42)	151 (21.88)	
Provider preference expressed at baseline [1203]^b^				
Specialist Provider	730 (60.68)	299 (58.28)	431 (62.46)	5.34
Non-Specialist Provider	22 (1.83)	14 (2.73)	8 (1.16)	
No Preference	451 (37.49)	200 (38.99)	251 (36.38)	

^**^p < 0.01, ^***^p < 0.001.

^a^Four participants did not complete the baseline assessment. ^b^This question was added after the trial commencement (in Spring 2020). ^c^This question is a sub-question of another question.

^Δ^All Canadian participants have government insurance; some also have supplemental private insurance.

^BIPOC included Asian, Pacific Islander, Mixed race, Black/African American, Hispanic, American Indian/Alaska Native and Middle Eastern.

### Enrollment and retention rates

There was no statistically significant difference in monthly enrollment rates between groups (Peak: 25.38 vs. Non-Peak: 21.78 participants per month, *t*-value = -1.16, *p* = 0.250). Similarly, a chi-square test of independence revealed no significant relationship between participant retention during treatment and the COVID-19 periods (Peak: 98.12% vs. Non-peak: 98.42%, χ² = 0.160, *p* = 0.689). Logistic regression analysis, accounting for covariates, also showed no significant effect of the peak versus non-peak COVID intervals during treatment (*p* = 0.383). However, there was a significant difference between “retention until treatment” based on the COVID period (Peak: 96.44% vs. Non-peak: 98.42%, χ² = 5.01, *p* = 0.025). This means that a lower percentage of enrolled “peak” participants never started therapy. When controlling for covariates, the logistic regression evaluating retention until treatment did not show significance (*p* = 0.131).

### Symptom scores

No statistically significant differences were observed in depressive or anxiety symptoms between the peak and non-peak COVID periods at both baseline (EPDS: 15.85 vs. 15.72, *p* = 0.546; GAD-7: 11.70 vs. 11.96, *p* = 0.3670) and 3- months post-randomization (EPDS: 9.09 vs. 9.09, *p* = 0.980; GAD-7: 6.42 vs. 6.38, *p* = 0.907) (**Table 2**).

**Table 2:** Mean depressive and anxiety symptom scores at baseline and 3 months for full cohort and peak and non-peak subgroups (N = 1230).

Variable (range), mean (95% CI)	Baseline (N = 1230)			3-months (n=1098)				
	Overall (N = 1230)	Peak (n=533)	Non-peak (n=697)	*t*-value	Overall (n=1098)	Peak (n=473)	Non-peak (n=625)	*t*-value
Depressive Symptoms (EPDS; 0-30)	15.77 (15.56- 15.99)	15.85 (15.51-16.19)	15.72 (15.44- 15.99)	-0.60	9.09 (8.79- 9.39)	9.09 (8.62-9.55)	9.09 (8.70- 9.49)	0.02
Anxiety Symptoms (GAD-7; 0-21)	11.85 (11.58- 12.12)	11.70 (11.26-12.15)	11.96 (11.62-12.31)	0.91	6.40 (6.10- 6.70)	6.42 (5.95-6.89)	6.38 (6.00- 6.77)	-0.12

EPDS, Edinburgh Postnatal Depression Scale; GAD, Generalized Anxiety Disorder (GAD-7).

### Participant satisfaction

There was no statistically significant difference in participant satisfaction (CSQ-8) scores between peak and non-peak periods (CSQ-8: 3.45 vs. 3.39, *p* = 0.174). Nor did linear mixed model analyses reveal a significant effect of the COVID period on patient satisfaction score (β = 0.05, *p* = 0.304), after controlling for relevant covariates. Only 1033 patients out of the full cohort had at least one treatment session and completed CSQ. Data was imputed for individuals (n=86) who completed sessions but did not complete the CSQ.

### Barriers and facilitators to positive care experiences

**Figure 2:**
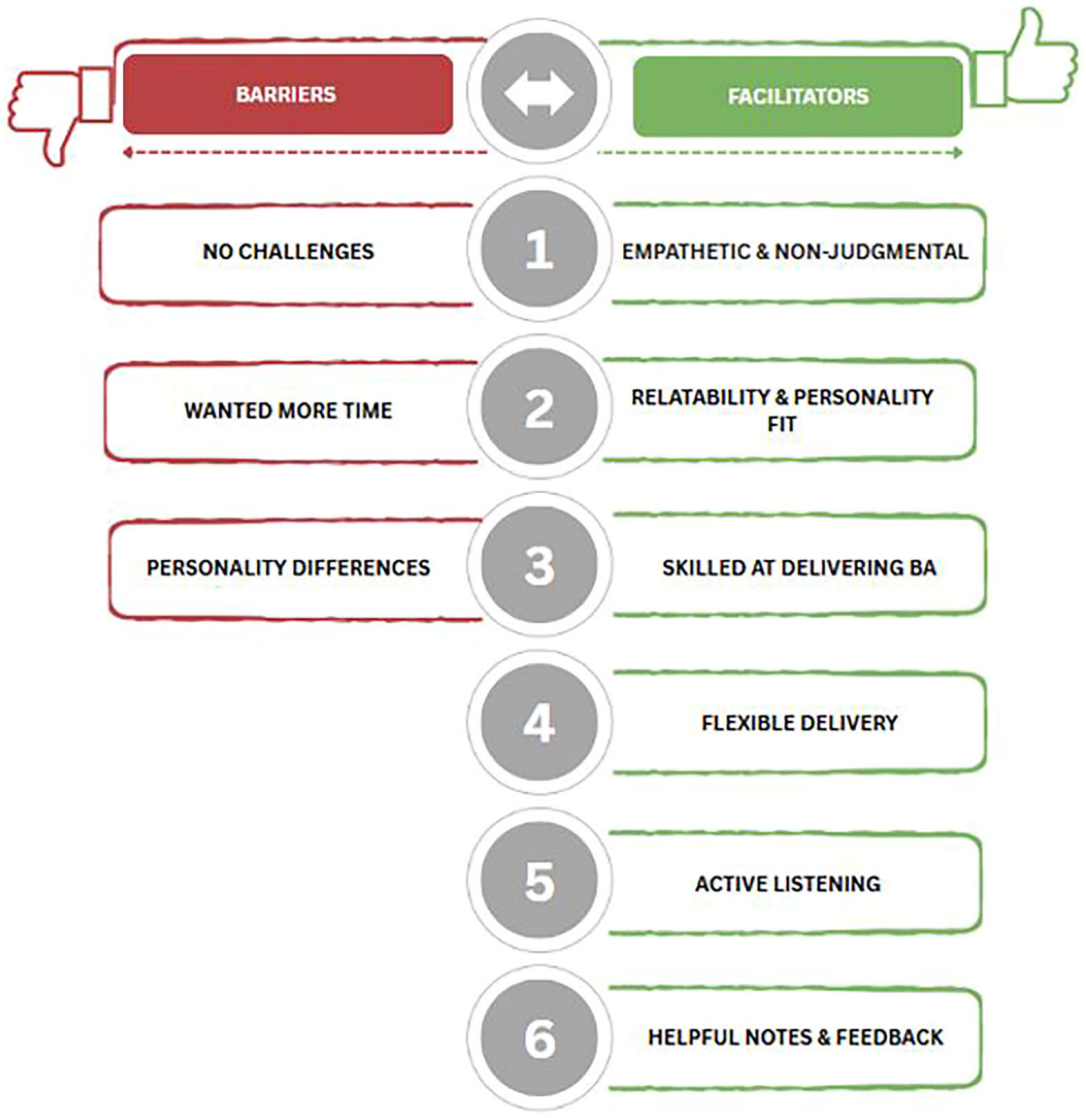
Barriers & facilitator categories to treatment satisfaction. **Figure 2** display the barriers and facilitators treatment reported by peak and non-peak perinatal participants. The same barriers and facilitators were reported by both groups. When asked about barriers, “no barriers” was reported most often by both groups.

The qualitative sample (N = 37) consisted of 19 peak and 18 non-peak perinatal participants (for full list of characteristics, see **Supplementary Table 3**). All reported facilitators and barrier categories are displayed in **Figure 2** and the breakdown of these categories are displayed in **Table 3**. Participants reported similar perceived *facilitators* to satisfactory care experiences across both periods. These facilitators included: (1) empathetic and non-judgmental providers (Peak n=8, 42.0%; Non-peak n=12, 66.7%), (2) relatable and compatible providers (Peak n=8, 42.0%; Non-peak n=11, 61.1%), (3) providers skilled at delivering BA (Peak n=7, 37.0%; Non-peak n=12, 66.7%), (4) flexible (Peak n=5, 26.0%; Non-peak n=8, 44.4%), (5) skilled at active listening (Peak n=5, 26.0%; Non-peak n=5, 27.8%), and (6) provided helpful notes and feedback (Peak n=2, 11.0%; Non-peak n=6, 33.3%). Participants in the non-peak period endorsed each facilitator more frequently, which may have been shaped by their enrollment in in-person sessions due to COVID or COVID-19 pandemic itself. Nonetheless, as described, there was no statistical difference in treatment satisfaction among participants from both groups.

**Table 3:** Barriers and facilitators to treatment by peak and non-peak COVID subgroups (n=37).

Key Themes (n=37)	Peak (n=19, 51.35%)	Non-peak (n=18, 48.65%)
Facilitators		
Empathetic & Non-Judgmental	8 (42.0)	12 (66.7)
Relatability & Personality Fit	8 (42.0)	11 (61.1)
Skilled at Delivering BA	7 (37.0)	12 (66.7)
Flexible Delivery	5 (26.0)	8 (44.4)
Active Listening	5 (26.0)	5 (27.8)
Helpful Notes & Feedback	2 (11.0)	6 (33.3)
Barriers		
Wanted More Time	3 (16.0)	5 (27.8)
Personality Differences	3 (16.0)	2 (11.1)

Representative facilitator comments included those that reflected favorably on the NSP’s relatability as a mother:

“[My provider] also happens to be a mother and has a young child. I felt like that was helpful to me, and was particularly affirming … because they also identify as a woman, there were definite moments when I ranted and then at the end just kind of went ‘well, patriarchy’ [Laughter]. It was nice, they understood and or at least laughed a little bit because some of my observations, she said, resonated with her as well.” (Sinai Participant 61, Non-peak COVID)

and the acknowledgement that the participant was not the only individual with emotional challenges associated with childbirth:

“It was helpful to have [a provider] who is a mom [since] my struggle really stemmed from challenges with breastfeeding … She was really relatable. I’ve been in therapy before and I felt like I haven’t had that same connection with any other therapists.” (Northshore Participant 2, Peak COVID)

When asked about *barriers* to satisfactory treatment, both peak and non-peak participants reported few barriers that were similar in frequency between peak and non-peak groups. The most common “barrier” was participants’ desire for more sessions to develop a better connection with their provider (Peak n=3, 16.0%; Non-peak n=5, 27.8%):

“I only have good things to say about [provider’s name]. One thing was craving for more time with her [laughter]. I would have liked our sessions to be longer. It’s not feedback on her by any means. I feel a little bit more time could have really enhanced the quality of the conversations.” (Sinai Participant 70, Non-peak COVID)

Overall, it did not appear that COVID-19 peak periods were associated with unique facilitators or barriers and the impact of telemedicine during high COVID-19 prevalence may have accounted for these observations.

### Telemedicine-only cases

In order to control for the potential impact of treatment modality (i.e., in-person treatment) that was only assigned during the non-peak-COVID-19 timeframe, we conducted a sub-analysis excluding the non-peak, in-person cases, resulting in 533 and 408 participants, in the peak and non-peak periods, respectively (**Table 4**).

**Table 4:** Baseline characteristics categorized for telemedicine-only cohort by COVID phases, Peak and Non-peak groups (n=941).

Variable [Telemedicine-only]	Overall (n=941)	Peak (n=533)	Non-peak (n=408)	*t-value/χ^2^*
Age (years) [937], mean (95% CI)^a^	33.06 (32.74-33.38)	32.79 (32.37-33.21)	33.41 (32.93-33.90)	1.92
Perinatal period [941]				
Pregnant	477 (50.69)	277 (51.97)	200 (49.02)	0.80
Postpartum	464 (49.31)	256 (48.03)	208 (50.98)	
Race (White vs. BIPOC^^^) [937] ^a^				
White	473 (50.48)	295 (55.77)	178 (43.63)	15.41^**^
Racial and ethnic minorities	437 (46.64)	217 (41.02)	220 (53.92)	
Prefer not to answer	27 (2.88)	17 (3.21)	10 (2.45)	
Born in country of your current residence? [937]^a^	653 (69.69)	380 (71.83)	273 (66.91)	7.18^*^
Health Benefits Access^Delta;^ [671]^b^	466 (69.45)	198 (72.26)	268 (67.51)	2.43
Marital status [937]^a^				
Married or Stable relationship	806 (86.02)	460 (86.96)	346 (84.80)	1.17
Single	71 (7.58)	36 (6.81)	35 (8.58)	
Dating or Uncommitted relationship	31 (3.31)	17 (3.21)	14 (3.43)	
Other	14 (1.49)	8 (1.51)	6 (1.47)	
Prefer not to answer	15 (1.60)	8 (1.51)	7 (1.72)	
Gender identity [886]^b^				
Female	881 (99.44)	476 (99.58)	405 (99.26)	1.20
Sexual orientation [886]^b^				
Straight/Heterosexual	810 (91.42)	438 (91.63)	372 (91.18)	1.49
Bisexual	52 (5.87)	25 (5.23)	27 (6.62)	
Other	6 (0.68)	4 (0.84)	2 (0.49)	
Prefer not to answer	18 (2.03)	11 (2.30)	7 (1.72)	
Highest level of education [937]^a^				
University degree	655 (69.90)	373 (70.51)	282 (69.12)	4.56
College/Trade School	161 (17.18)	89 (16.82)	72 (17.65)	
High School or less	112 (11.95)	65 (12.29)	47 (11.52)	
Prefer not to answer	9 (0.96)	2 (0.38)	7 (1.72)	
Employment [937]^a^				
Full-time	366 (39.06)	204 (38.56)	162 (39.71)	4.32
Maternity leave	268 (28.60)	142 (26.84)	126 (30.88)	
Part-time	83 (8.86)	47 (8.88)	36 (8.82)	
Not employed	207 (22.09)	129 (24.39)	78 (19.12)	
Other	13 (1.39)	7 (1.32)	6 (1.47)	
Nulliparous[937]^a^	524 (55.92)	293 (55.39)	231 (56.62)	2.98
COVID Exposure [787]^b^	22 (2.79)	2 (0.51)	20 (5.03)	14.90^***^
Self-Reported History of Depression/Anxiety [937]^a^	805 (85.91)	460 (86.96)	345 (84.56)	3.02
Related to current Baby	248 (30.81)	139 (30.22)	109 (31.59)	1.56
Dosage[887]	6.95 (6.82-7.08)	7.04 (6.88-7.20)	6.84 (6.63-7.04)	-1.53
Psychotropic medication Use at Baseline [227]^c^	227 (100)	133 (100)	94 (100)	–
Pregnancy conditions during current pregnancy [798] ^b^				
Preeclampsia	36 (4.51)	18 (4.49)	18 (4.49)	0.35
High blood-pressure	82 (10.28)	46 (11.47)	36 (9.07)	1.60
Gestational diabetes	85 (10.65)	46 (11.47)	39 (9.82)	0.57
Preterm labor	51 (6.39)	25 (6.23)	26 (6.55)	0.73
Treatment preference expressed at baseline [918]^b^				
Telemedicine	595 (64.81)	336 (65.50)	259 (63.95)	0.37
In-Person	116 (12.64)	62 (12.09)	54 (13.33)	
No Preference	207 (22.55)	115 (22.42)	92 (22.72)	
Provider preference expressed at baseline [918]^b^				
Specialist Provider	552 (60.13)	299 (58.28)	253 (62.47)	2.79
Non-Specialist Provider	20 (2.18)	14 (2.73)	6 (1.48)	
No Preference	346 (37.69)	200 (38.99)	146 (36.05)	

^**^p < 0.01, ^***^p < 0.001.

*Note.*^a^Four participants did not complete the baseline assessment. ^b^This question was added after the trial commencement (in Spring 2020). ^c^This question is a sub-question of another question.

^Delta;^All Canadian participants have government insurance; some also have supplemental private insurance.

^BIPOC included Asian, Pacific Islander, Mixed race, Black/African American, Hispanic, American Indian/Alaska Native and Middle Eastern.

In this analysis, a Chi-square test of independence revealed no significant relationship between participant retention during treatment and the COVID-19 periods (Peak: 98.12% vs. Non-peak: 97.30%, χ² = 0.712, *p* = 0.399). Additionally, this cohort exhibited no significant differences in the depression and anxiety symptom scores at baseline (EPDS: Peak: 15.85 vs. Non-peak: 15.73, *p* = 0.619; GAD-7: Peak: 11.70 vs. Non-peak: 11.98, *p* = 0.384) and 3-months post-randomization (EPDS: Peak: 9.09 vs. Non-peak: 9.22, *p* = 0.710; GAD-7: Peak: 6.42 vs. Non-peak: 6.45, *p* = 0.929) (**Table 5**).

**Table 5:** Depressive and anxiety symptom scores at baseline and 3 months for telemedicine-only cohort and peak and non-peak subgroups (n=941).

Variable (range), mean (95% CI)	Baseline (n=941)				3-months (n=836)			
	Overall (n=941)	Peak (n=533)	Non-peak (n=408)	*t-*value	Overall (n=836)	Peak (n=473)	Non-peak (n=363)	*t-*value
Depressive Symptoms (EPDS; 0-30)	15.80 (15.55- 16.04)	15.85 (15.51-16.19)	15.73 (15.37- 16.08)	-0.50	9.15 (8.79-9.50)	9.09 (8.62-9.55)	9.22 (8.67- 9.78)	0.37
Anxiety Symptoms (GAD-7; 0-21)	11.82 (11.51- 12.14)	11.70 (11.26-12.15)	11.98 (11.54- 12.42)	0.87	6.43 (6.09- 6.78)	6.42 (5.95-6.89)	6.45 (5.94- 6.96)	0.09

EPDS, Edinburgh Postnatal Depression Scale; GAD, Generalized Anxiety Disorder (GAD-7).

Finally, there was no statistically significant difference in participant satisfaction (CSQ-8) scores between peak and non-peak periods for the telemedicine-only group (CSQ-8: 3.45 vs. 3.41, *p* = 0.349), and Linear mixed model analyses revealed no significant effect of the COVID period on patient satisfaction score (β = 0.05, *p* = 0.297), after controlling for relevant covariates. Only 816 patients out of the telemedicine cohort had at least one treatment session and completed CSQ. Data was imputed for individuals (n=71) who completed sessions but did not complete the CSQ.

## Discussion

The COVID-19 pandemic provided a unique opportunity to understand if the high infection prevalence periods had a negative impact on the measured outcomes for SUMMIT participants. The current study used a mixed methods approach to compare patient engagement and treatment efficacy during peak and non-peak COVID-19 timeframes corresponding to the adaptations put in place to continue SUMMIT safely. Specifically, we examined treatment engagement via rates of participant enrollment, retention during therapy, as well as treatment satisfaction; assessed treatment outcomes via changes in depressive and anxiety symptom scores. We also examined perceived barriers and facilitators of care experiences among a subset of interviewed participants. Our findings contribute to our understanding of how healthcare utilization and satisfaction with treatment may vary during public health crises and underscore the importance of adaptive care delivery models in response to unanticipated public health disruptions.

### Telemedicine in times of crisis: maintaining engagement during COVID-19

Enrollment rates in the SUMMIT Trial were similar and stable when comparing peak and non-peak periods. This suggests that the pandemic’s peak did not deter individuals from seeking care once the remote accommodation was put in place. This finding contrasts with other studies reporting challenges in enrollment during the pandemic (31, 32). A global review of clinical trial activity across 37 countries reported a 25-50% decline in in-person enrollment across multiple therapeutic areas between 2019 and 2021 (33). In mental healthcare, telemedicine may help address such challenges by offering a safer and more convenient alternative to in-person care, particularly during times of public health disruption (34). This modality is especially relevant in the context of low enrollment among racial and ethnic minority populations in clinical trials during the pandemic, which coincided with a disproportionate burden of COVID-19-related health and social impacts in these communities (35, 36). Notably, modality adaptations to treatment delivery have been linked to equally high satisfaction rates among racial and ethnic minorities compared to White participants (15). Expanding access to telemedicine-based services may mitigate some barriers by increasing flexibility and reducing logistical and systemic obstacles to participation and care.

Retention rates during treatment in the SUMMIT Trial also remained stable, with no significant differences observed between peak and non-peak periods. This suggests consistent participant engagement once treatment commenced, despite external stressors associated with the pandemic. These findings align with existing literature, which highlights an increased preference for telemedicine among providers and patients during the pandemic (37) and thereafter (38). This growing acceptance of telehealth practices has been associated with higher attendance rates and fewer cancellations across various healthcare domains (39, 40), including among perinatal populations. Similarly, other clinical trials conducted during the COVID-19 pandemic achieved high retention rates, often exceeding 80%, by implementing telemedicine and online health interventions—rates comparable to or higher than those observed in pre-pandemic, in-person trials (41–44). Although there were no observable differences in retention across peak and non-peak periods, there were sociodemographic differences, particularly that peak period participants were more likely to be younger and identify as White than non-peak participants. The difference in age between peak and non-peak participants was minimal (approximately 1 year), and the statistical significance is likely attributable to the large sample size rather than a clinically meaningful difference. Although speculative, differences in the racial makeup of peak vs non-peak enrollment could be related to a few different factors: participants identifying as racial or ethnic minorities may have been more likely to live with extended family (45) and therefore have less private space for virtual visits, be less reliant on outside childcare when school was conducted virtually and therefore have less time to enroll in a research study, and/or have differences in the ability and sources to work virtually (46, 47) that may have impacted enrollment during the telemedicine only periods.

No significant differences in participant satisfaction were observed across COVID-19 periods, with satisfaction remaining consistently high throughout the pandemic. This finding aligns with existing research suggesting that telemedicine-delivered care effectively maintains high levels of treatment satisfaction, upwards of 80% (48). The high satisfaction rates observed in the SUMMIT Trial may also be attributed to the implementation of behavioral activation that was also culturally sensitive, which aimed to strengthen the therapeutic relationship by incorporating the requisite humility to facilitate discussions about race, culture, and ethnicity (15). These findings reflect the SUMMIT model’s patient-centered orientation, which prioritized personalized care. The emphasis on empathy, flexibility, and cultural humility makes participants feel heard and respected.

### Exploring treatment outcomes and participant experiences during COVID-19

No statistically significant differences in baseline as well as 3-month measures of depressive or anxiety symptoms were observed in either cohort. These stable symptom trajectories may be attributable to the high and uniform treatment fidelity (5) achieved across modalities (Telemedicine and In-person) and provider (Non-specialist vs. Specialist) groups (as shown in our previous publication), which likely helped maintain intervention effectiveness under pandemic-related stressors. However, these findings diverge from previous literature suggesting that the COVID-19 pandemic exacerbated the prevalence of perinatal depression and anxiety symptoms up to 24.9% and 32.8% respectively (49). This discrepancy may reflect the effectiveness of proactive and supportive care models like SUMMIT (5) in buffering against the adverse mental health effects typically associated with the pandemic. Participants in this study may have benefited from early intervention, consistent access to care via telemedicine, and providers trained in delivering empathetic, evidence-based interventions—all of which could mitigate symptom exacerbation. Furthermore, the structured support offered through SUMMIT may have fostered a sense of continuity and safety during an otherwise unpredictable period, potentially offsetting broader population-level trends reported in other studies. Thus, care models that prioritize provider well-being, patient well-being, and the integration of telemedicine represent promising strategies for expanding access to care in both pandemic and non-pandemic contexts (50–52).

The study’s qualitative data confirmed that SUMMIT provided needed support and a satisfying therapeutic experience to participants, independent of the pandemic. Participants from both peak and non-peak periods identified similar facilitators to treatment delivery, including empathetic and non-judgmental care, compatibility with their provider, effective delivery of BA therapy, flexibility in scheduling, active listening, and thoughtful feedback. Non-peak participants more frequently endorsed these facilitators, potentially reflecting improvements in provider experience and the refinement of telemedicine practices over time. This interpretation aligns with existing literature, which suggests that effective treatment delivery may improve as providers gain technological competence and experience with remote care (53). Barriers to treatment delivery were reported less frequently than facilitators, and types of barriers were consistently reported across both periods. This trend mirrors research that indicates barriers to treatment delivery during the pandemic were less frequently reported than facilitators, while the types of barriers and facilitators remained consistent (54–56).

### Strengths and limitations

This study has several key strengths. First, our participant sample included varied racial and ethnic representation, enhancing our findings’ generalizability (57). This is particularly important given the disproportionate impact of the COVID-19 pandemic on racial and ethnic minorities (36, 58) and the historic lack of effective engagement of treatment studies with these populations (59). Second, including in-person participants in our analyses mitigated the risk of selection bias and provided critical context for understanding enrollment patterns across the various phases of the pandemic. Finally, combining quantitative assessments with qualitative data strengthened our understanding of the participants’ experience of receiving mental health care during the pandemic (60).

Our study also has limitations. First, participants who enrolled but did not receive any treatment were unavailable for qualitative interviews. Thus, we cannot determine if the dropout was specific to the pandemic which could modify our conclusions. Second, no analyses were done to account for the potential variation in treatment outcomes between earlier and later peak and non-peak COVID periods. It is possible that improvements in outcomes over time may have reflected increased familiarity with telemedicine, refined clinical workflows, or greater comfort among both providers and participants. Future studies should aim to include potential confounders such as seasonal variations, social support availability, shifting medical and safety concerns and their impact on the study outcomes. These variables were not collected for this study. The studies should also include perspectives from individuals who enrolled but did not initiate treatment to better understand barriers to engagement, particularly in the context of external stressors like a pandemic. Additionally, the temporal trends in treatment outcomes across different phases of the COVID were not assessed due to small sample sizes across various phases, which would not have yielded meaningful results (61). However, future research should explore this to ensure more comprehensive estimates of treatment outcomes. This could help disentangle the effects of evolving contextual factors—such as increasing telehealth proficiency, shifting public health guidelines, and changing participant attitudes—on both engagement and clinical effectiveness.

## Conclusions

Adjustment of the SUMMIT trial protocol to the telemedicine only condition in response to the COVID-19 pandemic allowed trial recruitment and treatment to continue during times of peak infection. We observed that clinical engagement (as measured by rates of participant enrollment, retention, and satisfaction), efficacy (as measured by depressive and anxiety symptom scores), and positive participant experiences was preserved throughout the peak and non-peak pandemic timeframes. This study provides key insights into maintaining mental health treatment during public health emergencies that otherwise prevent in-person care.

## Data Availability

The datasets generated and/or analyzed during the current study are not publicly available to allow the study team to collect long-term study outcomes and analyze and potentially publish the results of the secondary aims involving sustained outcomes at 12-months post-randomization as indicated in our detailed Statistical Analysis Plan, but are available from the corresponding author on reasonable request. Requests to access the datasets should be directed to Daisy Singla, daisy.singla@utoronto.ca.
