# Supplementary material for: Engagement, efficacy, and experiences of psychotherapy for perinatal populations with depression and anxiety during the COVID-19 pandemic
Source: Front Psychiatry. 2026 Jan 22;16:1686719. doi: 10.3389/fpsyt.2025.1686719 (PMC12875091; doi:10.3389/fpsyt.2025.1686719)
Supplement: Supplementary file 1 [file Table1.docx]

# Supplementary Table 1**:** Baseline Participant Characteristics Categorized by Peak and Non-peak COVID-19 Timeframes for those who Completed 3-month Post-randomization Assessment (N=1098)

| **Variable [Total Respondents]** | **Overall**  **(n=1098)** | **Peak COVID**  **(n=473)** | **Non-peak COVID**  **(n=625)** | ***t-value/χ^2^*** |
| --- | --- | --- | --- | --- |
| **Age** (years) [1098], mean (95% CI) | 33.42 (33.13-33.70) | 32.95 (32.51-33.39) | 33.76 (33.38-34.15) | 2.74^**^ |
| **Perinatal Period** [1098] | | | | |
| Pregnant | 548 (49.91) | 244 (51.59) | 304 (48.64) | 0.93 |
| Postpartum | 550 (50.09) | 229 (48.41) | 321 (51.36) |  |
| **Race (White vs. BIPOC**^^^**)** [1098] | | | | |
| White | 551 (50.18) | 266 (56.24) | 285 (45.60) | 13.19^**^ |
| Racial and ethnic minorities | 517 (47.09) | 193 (40.80) | 324 (51.84) |  |
| Prefer not to answer | 30 (2.73) | 14 (2.96) | 16 (2.56) |  |
| **Born in country of your current residence?** [1098] | 763 (69.49) | 336 (71.04) | 427 (68.32) | 0.6 |
| **Health Benefits Access**^∆^ [856]^a^ | 591 (69.04) | 184 (73.02) | 407 (67.38) | 3.38 |
| **Marital Status** [1098] | | | | |
| Married or Stable relationship | 951 (86.61) | 418 (88.37) | 533 (85.28) | 3.33 |
| Single | 82 (7.47) | 29 (6.13) | 53 (8.48) |  |
| Dating or Uncommitted relationship | 34 (3.1) | 14 (2.96) | 20 (3.2) |  |
| Other | 16 (1.46) | 5 (1.06) | 11 (1.76) |  |
| Prefer not to answer | 15 (1.37) | 7 (1.48) | 8 (1.28) |  |
| **Gender Identity** [1075]^a^ | | | | |
| Female | 1071 (99.63) | 449 (99.56) | 622 (99.68) | 2.16 |
| **Sexual Orientation [**1075]^a^ | | | | |
| Straight/Heterosexual | 987 (91.81) | 414 (91.8) | 573 (91.83) | 2.80 |
| Bisexual | 63 (5.86) | 23 (5.1) | 40 (6.41) |  |
| Other | 7 (0.65) | 4 (0.89) | 3 (0.48) |  |
| Prefer not to answer | 18 (1.67) | 10 (2.22) | 8 (1.28) |  |
| **Highest Level of Education** [1098] | | | | |
| University degree | 785 (71.49) | 347 (73.36) | 438 (70.08) | 3.33 |
| College/Trade School | 185 (16.85) | 72 (15.22) | 113 (18.08) |  |
| High School or less | 119 (10.84) | 52 (10.99) | 67 (10.72) |  |
| Prefer not to answer | 9 (0.82) | 2 (0.42) | 7 (1.12) |  |
| **Employment** [1098] | | | | |
| Full-time | 428 (38.98) | 179 (37.84) | 249 (39.84) | 3.20 |
| Maternity leave | 323 (29.42) | 134 (28.33) | 189 (30.24) |  |
| Part-time | 100 (9.11) | 42 (8.88) | 58 (9.28) |  |
| Not employed | 230 (20.95) | 111 (23.47) | 119 (19.04) |  |
| Other | 17 (1.55) | 7 (1.48) | 10 (1.6) |  |
| **Nulliparous**[1098] | 606 (55.19) | 267 (56.45) | 339 (54.24) | 0.35 |
| **COVID Exposure** [959]^a^ | 29 (3.02) | 2 (0.56) | 27 (4.47) | 11.64^***^ |
| **Self-Reported History of Depression/Anxiety** [1098] | 941 (85.70) | 413 (87.32) | 528 (84.48) | 2.09 |
| Related to the current Baby | 283 (30.07) | 127 (30.75) | 156 (29.55) | 0.63 |
| **Dosage**[1039] | 7.04 (6.93-7.15) | 7.25 (7.11-7.39) | 6.87 (6.70-7.04) | -3.39^***^ |
| **Psychotropic medication Use at Baseline**  [256]^b^ | 256 (100) | 122 (100) | 134 (100) | - |
| **Pregnancy conditions during current pregnancy** [967]^a^ | | | | |
| Preeclampsia | 48 (4.96) | 17 (4.68) | 31 (5.13) | 0.11 |
| High blood-pressure | 112 (11.58) | 41 (11.29) | 71 (11.75) | 0.05 |
| Gestational diabetes | 103 (10.65) | 38 (10.47) | 65 (10.76) | 0.02 |
| Preterm labor | 60 (6.20) | 23 (6.34) | 37 (6.13) | 0.02 |
| **Treatment Preference Expressed at Baseline** [1084]^a^ | | | | |
| Telemedicine | 673 (62.08) | 308 (66.38) | 365 (58.87) | 8.68^*^ |
| In-Person | 170 (15.68) | 57 (12.28) | 113 (18.23) |  |
| No Preference | 241 (22.23) | 99 (21.34) | 142 (22.9) |  |
| **Provider Preference Expressed at Baseline** [1084]^a^ | | | | |
| Specialist Provider | 655 (60.42) | 267 (57.54) | 388 (62.58) | 5.94 |
| Non-Specialist Provider | 20 (1.85) | 13 (2.8) | 7 (1.13) |  |
| No Preference | 409 (37.73) | 184 (39.66) | 225 (36.29) |  |

^*^*p*<0.05, ^**^*p*<0.01, ^***^*p*<0.001

*Note.* ^a^This question was added after the trial commencement (in Spring 2020). ^b^This question is a sub-question of another question.

^∆^All Canadian participants have government insurance and some also have supplemental private insurance.

^BIPOC included Asian, Pacific Islander, Mixed race, Black/African American, Hispanic, American Indian/Alaska Native and Middle Eastern.

# Supplementary Table 2**:** Baseline Participant Characteristics Categorized by Peak and Non-peak COVID-19 Timeframes for those who did not Complete 3-month post-randomization assessment (N=132)

| **Variable [Total Respondents]** | **Overall**  **(n=132)** | **Peak COVID=1**  **(n=60)** | **Non-peak COVID=0**  **(n=72)** | ***t-value/χ^2^*** |
| --- | --- | --- | --- | --- |
| **Age** (years) [128], mean (95% CI)[^a^](#explanationformissingdata) | 32.07 (31.13-33.01) | 31.39 (30.02-32.76) | 32.60 (31.30-33.89) | 1.26 |
| **Perinatal Period** [132] | | | | |
| Pregnant | 70 (53.03) | 33 (55.00) | 37 (51.39) | 0.17 |
| Postpartum | 62 (46.97) | 27 (45.00) | 35 (48.61) |  |
| **Race (White vs. BIPOC**^^^**)** [128][^a^](#explanationformissingdata) | | | | |
| White | 63 (49.22) | 29 (51.79) | 34 (47.22) | 0.34^#^ |
| Racial and ethnic minorities | 61 (47.66) | 24 (42.86) | 37 (51.39) |  |
| Prefer not to answer | 4 (3.13) | 3 (5.36) | 1 (1.39) |  |
| **Born in country of your current residence?** [128][^a^](#explanationformissingdata) | 97 (75.78) | 44 (78.57) | 53 (73.61) | 0.27 |
| **Health Benefits Access** [92][^b^](#explanationformissingdata) | 63 (68.48) | 14 (63.64) | 49 (70.00) | 0.33 |
| **Marital Status** [128][^a^](#explanationformissingdata) | | | | |
| Married or Stable relationship | 101 (78.91) | 42 (75) | 59 (81.94) | 0.75^#^ |
| Single | 12 (9.38) | 7 (12.5) | 5 (6.94) |  |
| Dating or Uncommitted relationship | 7 (5.47) | 3 (5.36) | 4 (5.56) |  |
| Other | 5 (3.91) | 3 (5.36) | 2 (2.78) |  |
| Prefer not to answer | 3 (2.34) | 1 (1.79) | 2 (2.78) |  |
| **Gender Identity** [98][^b^](#explanationformissingdata) | | | | |
| Female | 97 (98.98) | 27 (100.00) | 70 (98.59) | 1.00^#^ |
| **Sexual Orientation [**98][^b^](#explanationformissingdata) | | | | |
| Straight/Heterosexual | 86 (87.76) | 24 (88.89) | 62 (87.32) | 1.00^#^ |
| Bisexual | 8 (8.16) | 2 (7.41) | 6 (8.45) |  |
| Other | 0 (0.00) | 0 (0.00) | 0 (0.00) |  |
| Prefer not to answer | 4 (4.08) | 1 (3.70) | 3 (4.23) |  |
| **Highest Level of Education** [128][^a^](#explanationformissingdata) | | | | |
| University degree | 74 (57.81) | 26 (46.43) | 48 (66.67) | 0.057^#^ |
| College/Trade School | 31 (24.22) | 17 (30.36) | 14 (19.44) |  |
| High School or less | 22 (17.19) | 13 (23.21) | 9 (12.5) |  |
| Prefer not to answer | 1 (0.78) | 0 (0.00) | 1 (1.39) |  |
| **Employment** [128][^a^](#explanationformissingdata) | | | | |
| Full-time | 56 (43.75) | 25 (44.64) | 31 (43.06) | 0.088^#^ |
| Maternity leave | 30 (23.44) | 8 (14.29) | 22 (30.56) |  |
| Part-time | 11 (8.59) | 5 (8.93) | 6 (8.33) |  |
| Not employed | 30 (23.44) | 18 (32.14) | 12 (16.67) |  |
| Other | 1 (0.78) | 0 (0.00) | 1 (1.39) |  |
| **Nulliparous**[128][^a^](#explanationformissingdata) | 62 48.44 | 26 (46.43) | 36 (50.00) | 0.23 |
| **COVID Exposure** [105][^b^](#explanationformissingdata) | 2 (1.90) | 0 (0.00) | 2 (2.86) | 0.55^#^ |
| **Self-Reported History of Depression/Anxiety** [128][^a^](#explanationformissingdata) | 110 (85.94) | 47 (83.93) | 63 (87.50) | 0.62 |
| Related to the current Baby | 38 (34.55) | 12 (25.53) | 26 (41.27) | 3.40 |
| **Dosage**[80] | 3.95 (3.40-4.50) | 4.15 (3.27-5.02) | 3.80 (3.06-4.54) | -0.61 |
| **Psychotropic medication Use at Baseline**  [32][^c^](#explanationformissingdata) | 32 (100) | 11 (100) | 21 (100) | - |
| **Pregnancy conditions during current pregnancy** [108][^b^](#explanationformissingdata) | | | | |
| Preeclampsia | 4 (3.70) | 1 (2.63) | 3 (4.29) | 1.00^#^ |
| High blood-pressure | 15 (13.89) | 5 (13.16) | 10 (14.29) | 0.04 |
| Gestational diabetes | 18 (16.67) | 8 (21.05) | 10 (14.29) | 0.75 |
| Preterm labor | 9 (8.33) | 2 (5.26) | 7 (10.00) | 0.48^#^ |
| **Treatment Preference Expressed at Baseline** [119][^b^](#explanationformissingdata) | | | | |
| Telemedicine | 74 (62.18) | 28 (57.14) | 46 (65.71) | 7.88^*^ |
| In-Person | 20 (16.81) | 5 (10.20) | 15 (21.43) |  |
| No Preference | 25 (21.01) | 16 (32.65) | 9 (12.86) |  |
| **Provider Preference Expressed at Baseline** [119][^b^](#explanationformissingdata) | | | | |
| Specialist Provider | 75 (63.03) | 32 (65.31) | 43 (61.43) | 0.85^#^ |
| Non-Specialist Provider | 2 (1.68) | 1 (2.04) | 1 (1.43) |  |
| No Preference | 42 (35.29) | 16 (32.65) | 26 (37.14) |  |

^*^*p*<0.05, ^**^*p*<0.01, ^***^*p*<0.001

*Note.* ^a^Four participants did not complete the baseline assessment. ^b^This question was added after the trial commencement (in Spring 2020). ^c^This question is a sub-question of another question.

^∆^All Canadian participants have government insurance and some also have supplemental private insurance.

^#^ Reported Fishers exact *p*-values.

^BIPOC included Asian, Pacific Islander, Mixed race, Black/African American, Hispanic, American Indian/Alaska Native and Middle Eastern.

# Supplementary Table 3: Baseline Participant Characteristics Categorized by Peak and Non-peak COVID-19 Timeframes for those who Completed Qualitative Interviews (N=37)

| **Variable [Total Respondents]** | **Overall**  **(n=37)** | **Peak COVID**  **(n=19)** | **Non-peak COVID**  **(n=18)** | ***t-value/χ^2^*** |
| --- | --- | --- | --- | --- |
| **Age** (years) [37], mean (95% CI) | 33.76 (32.18-35.34) | 32.68 (30.17-35.20) | 34.89 (32.90-36.88) | 1.44 |
| **Perinatal Period** [37] | | | | |
| Pregnant | 15 (40.54) | 7 (36.84) | 8 (44.44) | 0.22 |
| Postpartum | 22 (59.46) | 12 (63.16) | 10 (55.56) |  |
| **Race (White vs. BIPOC**^^^**)** [37] | | | | |
| White | 17 (45.95) | 9 (47.37) | 8 (44.44) | 0.51^#^ |
| Racial and ethnic minorities | 18 (48.65) | 8 (42.11) | 10 (55.56) |  |
| Prefer not to answer | 2 (5.41) | 2 (10.53) | 0 (0.00) |  |
| **Born in country of your current residence?** [37] | 23 (62.16) | 12 (63.16) | 11 (61.11) | 0.02 |
| **Health Benefits Access**^∆^ [21][^a^](#explanationformissingdata) | 19 (90.48) | 2 (66.67) | 17 (94.44) | 0.27^#^ |
| **Marital Status** [37] | | | | |
| Married or Stable relationship | 32 (86.49) | 17 (89.47) | 15 (83.33) | 0.69^#^ |
| Single | 3 (8.11) | 1 (5.26) | 2 (11.11) |  |
| Dating or Uncommitted relationship | 1 (2.7) | 0 (0.00) | 1 (5.56) |  |
| Other | 0 (0.00) | 0 (0.00) | 0 (0.00) |  |
| Prefer not to answer | 1 (2.7) | 1 (5.26) | 0 (0.00) |  |
| **Gender Identity** [37] | | | | |
| Female | 37 (100) | 19 (100) | 18 (100) | - |
| **Sexual Orientation [**37] | | | | |
| Straight/Heterosexual | 33 (89.19) | 18 (94.74) | 15 (83.33) | 0.33^#^ |
| Bisexual | 4 (10.81) | 1 (5.26) | 3 (16.67) |  |
| Other | 0 (0.00) | 0 (0.00) | 0 (0.00) |  |
| Prefer not to answer | 0 (0.00) | 0 (0.00) | 0 (0.00) |  |
| **Highest Level of Education** [37] | | | | |
| University degree | 29 (78.38) | 15 (78.95) | 14 (77.78) | 0.12^#^ |
| College/Trade School | 5 (13.51) | 1 (5.26) | 4 (22.22) |  |
| High School or less | 3 (8.11) | 3 (15.79) | 0 (0.00) |  |
| Prefer not to answer | 0 (0.00) | 0 (0.00) | 0 (0.00) |  |
| **Employment** [37] | | | | |
| Full-time | 13 (35.14) | 5 (26.32) | 8 (44.44) | 0.37^#^ |
| Maternity leave | 13 (35.14) | 8 (42.11) | 5 (27.78) |  |
| Part-time | 2 (5.41) | 2 (10.53) | 0 (0.00) |  |
| Not employed | 8 (21.62) | 4 (21.05) | 4 (22.22) |  |
| Other | 1 (2.7) | 0 (0.00) | 1 (5.56) |  |
| **Nulliparous**[37] | 19 (51.35) | 10 (52.63) | 9 (50.00) | 0.03 |
| **COVID Exposure** [30][^a^](#explanationformissingdata) | 1 (3.33) | 0 (0.00) | 1 (5.56) | 1.00^#^ |
| **Self-Reported History of Depression/Anxiety** [37] | 30 (81.08) | 16 (84.21) | 14 (77.78) | 0.69^#^ |
| Related to the current Baby | 10 (33.33) | 5 (31.25) | 5 (35.71) | 1.00^#^ |
| **Dosage**[37] | 7.73 (7.51-7.95) | 7.74 (7.42-8.05) | 7.72 (7.39-8.05) | -0.07 |
| **Psychotropic medication Use at Baseline** [10][^b^](#explanationformissingdata) | 10 (100) | 6 (100) | 4 (100) | - |
| **Pregnancy conditions during current pregnancy** [32][^a^](#explanationformissingdata) | | | | |
| Preeclampsia | 2 (6.25) | 0 (0.00) | 2 (11.11) | 0.49^#^ |
| High blood-pressure | 4 (12.50) | 1 (7.14) | 3 (16.67) | 0.61^#^ |
| Gestational diabetes | 3 (9.38) | 2 (14.29) | 1 (5.56) | 0.57^#^ |
| Preterm labor | 3 (9.38) | 1 (7.14) | 2 (11.11) | 1.00^#^ |
| **Treatment Preference Expressed at Baseline** [37] | | | | |
| Telemedicine | 27 (72.97) | 14 (73.68) | 13 (72.22) | 1.00^#^ |
| In-Person | 2 (5.41) | 1 (5.26) | 1 (5.56) |  |
| No Preference | 8 (21.62) | 4 (21.05) | 4 (22.22) |  |
| **Provider Preference Expressed at Baseline** [37] | | | | |
| Specialist Provider | 23 62.16 | 12 (63.16) | 11 (61.11) | 0.02 |
| Non-Specialist Provider | 0 (0.00) | 0 (0.00) | 0 (0.00) |  |
| No Preference | 14 37.84 | 7 (36.84) | 7 (38.89) |  |

^*^*p*<0.05, ^**^*p*<0.01, ^***^*p*<0.001

*Note.* ^a^This question was added after the trial commencement (in Spring 2020). ^b^This question is a sub-question of another question.

^∆^All Canadian participants have government insurance and some also have supplemental private insurance.

^#^ Reported Fishers exact *p*-values.

^BIPOC included Asian, Pacific Islander, Mixed race, Black/African American, Hispanic, American Indian/Alaska Native and Middle Eastern.

# **Appendix 1**

**SUMMIT Interview Guide for In-person BA Study Participants**

**Introduction:** Hello (insert name). My name is XXX and I am a qualitative researcher on the SUMMIT study team. Before we get started, I will share some information about the interview and this research. In this interview, you are invited to share your experiences participating in this study, and with receiving the SUMMIT Behavioural Activation (BA) treatment in-person. The experiences you share will help us understand how to facilitate better BA treatment in future to support others, and facilitate better training and supervision for providers. The interview will be about 30 to 45 minutes. As mentioned in the consent form, the interview will be audio recorded and transcribed. Your decision to take part in this interview is completely voluntary. You can end your participation at any time, just let me know. You do not need to answer any questions that make you feel uncomfortable, and you can feel free to stop me any time in case you have any questions for me. Also, there are no right or wrong answers to the questions because we are interested in your thoughts about how we can make the program better. If you decide to stop participating at any time, your decision will not influence the treatment you receive as a SUMMIT study participant. Do you have any questions? Is it ok to continue? Thank you, I will turn on the recorder now.

1. Do you currently have a preference for attending treatment (in-person or virtual)? If so, why?

a. Was Covid-19 a factor/ something you considered in your answer?

1. Have you been attending in-person BA? In general, how has your experience been with participating in IP BA during the pandemic?
2. Have there been any facilitators that have helped you or made it easier for you to attend in-person BA?
   1. What have you enjoyed about receiving therapy in-person?
   2. Have you noticed any benefits of attending in-person therapy?
   3. What did you not like about attending in-person therapy?
3. Building on things you did not like, have there been any challenges or barriers to attending the BA therapy in-person? If so, what has been challenging? (ie. Mask wearing, distancing, children not attending in-person school, etc.)
4. Is there anything that could have been done to support you better while attending in-person BA?
5. Do you think that there are particular sub-groups of women who may benefit more than others from in-person BA?
6. For hybrid participants: Were there any key differences in your experiences receiving BA virtually, vs in-person? If so, what are they?
